# Draft Genome Sequence of Plant Growth-Promoting Endophytic *Streptomyces* sp. GKU 895 Isolated from the Roots of Sugarcane

**DOI:** 10.1128/genomeA.00358-17

**Published:** 2017-05-11

**Authors:** Worarat Kruasuwan, Talal Sabhan Salih, Sarah Brozio, Paul A. Hoskisson, Arinthip Thamchaipenet

**Affiliations:** a Department of Genetics, Faculty of Science, Kasetsart University, Chatuchak, Bangkok, Thailand; b Strathclyde Institute of Pharmacy and Biomedical Sciences, University of Strathclyde, Glasgow, United Kingdom; c College of Science, University of Mosul, Mosul, Iraq; d Center for Advanced Studies in Tropical Natural Resources, NRU–KU, Kasetsart University, Chatuchak, Bangkok, Thailand

## Abstract

*Streptomyces* sp. GKU 895 is an endophytic actinomycete isolated from the roots of sugarcane. GKU 895 has a genome of 8.3 Mbp and the genome exhibits adaptations related to plant growth-promoting activity. It also has extensive specialized metabolite biosynthetic gene clusters apparent in its genome.

## GENOME ANNOUNCEMENT

Sugarcane is an economically important crop for the production of sugar and biofuels. Plant growth-promoting endophytes are heterogeneous groups of bacteria that reside mutually within plant tissues and appear to provide many benefits to the plant host (1). *Streptomyces* sp. GKU 895 is a root-associated bacterium isolated from sugarcane plants cultivated in Thailand (2). Based on 16S rRNA sequence analysis, strain GKU 895 is closely related to *Streptomyces canus* NRRLB-1989^T^ (99.4% similarity, GenBank accession no. KP637153). *Streptomyces* sp. GKU 895 shows the ability to solubilize phosphate and produce indole-3-acetic acid (IAA), siderophores, and 1-aminocyclopropane-1-carboxylate (ACC) deaminase. It is also capable of suppressing the growth of *Bacillus cereus* ATCC 11778 and the sugarcane red rot fungal pathogens *Colletotrichum falcatum* and *Fusarium moniliforme* (3). Moreover, *Streptomyces* sp. GKU 895 enhances sugarcane growth when applied in pot experiments (2). Here, we present the draft genome sequence of *Streptomyces* sp. GKU 895 determined using a combined sequencing approach using the Ion PGM and MinION.

Total DNA of *Streptomyces* sp. GKU 895 was extracted using an ISOLATE II genomic DNA extraction kit (BIOLINE, United Kingdom). The Ion PGM system produced 1,019,643 reads (with approximately 30× coverage) with an average read length of 240 bp. MinION reads were obtained using a ligation sequencing kit 2D (SQK-LSK208, R9.4) and spot-on flow cell Mk1 (R9.4) and were extracted using Poretools (4) which generated 21,275 2D MinION reads with an average length of 2,979 bp. The sequences were assembled using SPAdes version 3.9 with the nanopore option (5) and determined by QUAST version 3.2 (6). The input sequences were assembled to 190 contigs (coverage ≥10 and length ≥1,000) with an *N*_50_ of 61,010. The largest contig is 213,532 bp in length. The draft genome contains 8,296,413 bp with a G+C content of 70.7%.

The genome was annotated using the Rapid Annotations using Subsystems Technology (RAST) server (7), which identified 8,208 coding sequences. RNAmmer (8) and tRNAscan-SE (9) revealed 6 rRNA genes and 64 tRNA genes. RAST annotation revealed that the genome of *Streptomyces* sp. GKU 895 possesses genes associated with plant growth promotion including *acdS* gene-encoding ACC deaminase; genes involved in IAA synthesis [indoleacetamide hydrolase and nitrilase (10, 11)]; genes which assist in mineral phosphate solubilization including isocitrate dehydrogenase, citrate synthase, and purple acid phosphatase (12, 13); genes for the degradation of fungal cell walls [family 18 and 19 chitinases (14)]; and genes involved in host plant colonization such as salicylate hydroxylase (15). AntiSMASH (version 3.0) analysis (16) predicts 28 gene clusters involved in specialized metabolite production in the *Streptomyces* sp. GKU 895. These consist of six gene clusters encoding putative butyrolactones, three gene clusters of siderophores (including desferrioxamine E) and terpenes, two gene clusters for bacteriocins, melanins, type II polyketide synthases, and a single gene cluster encoding ectoine, nonribosomal peptide synthetase (NRPS), NRPS-bacteriocin, type III polyketide synthase (T3PKS), T3PKS-NRPS, T3PKS-terpene-butyrolactone, and terpene-butyrolactone-NRPS.

### Accession number(s).

The draft genome sequence of *Streptomyces* sp. GKU 895 has been deposited in the DDBJ/ENA/GenBank database under the accession number MWJO00000000.
